# Mosquito control exposures and breast cancer risk: analysis of 1071 cases and 2096 controls from the Ghana Breast Health Study

**DOI:** 10.1186/s13058-023-01737-x

**Published:** 2023-12-11

**Authors:** Naomie Olivos, Jim E. Banta, Rhonda Spencer-Hwang, Daniel Ansong, Laura E. Beane Freeman, Joe-Nat Clegg-Lamptey, Beatrice Wiafe-Addai, Lawrence Edusei, Ernest Adjei, Nicholas Titiloye, Florence Dedey, Francis Aitpillah, Joseph Oppong, Verna Vanderpuye, Ernest Osei-Bonsu, Thomas U. Ahearn, Richard Biritwum, Joel Yarney, Baffour Awuah, Kofi Nyarko, Montserrat Garcia-Closas, Mustapha Abubakar, Louise A. Brinton, Jonine D. Figueroa, Seth Wiafe

**Affiliations:** 1 School of Public Health, Loma Linda University, Loma Linda, CA USA; 2 Division of Cancer Epidemiology and Genetics, National Cancer Institute, Bethesda, MD USA; 3 Korle Bu Teaching Hospital, Accra, Ghana; 4 Peace and Love Hospital, Kumasi, Ghana; 5 Komfo Anokye Teaching Hospital, Kumasi, Ghana; 6 University of Ghana, Accra, Ghana; 7 Ministry of Health, Greater Accra Region, Ghana; 8 Institute for Cancer Research, London, UK

**Keywords:** Insecticide-treated nets, Breast cancer, Environmental exposure, Anti-mosquito interventions

## Abstract

Epidemiologic data on insecticide exposures and breast cancer risk are inconclusive and mostly from high-income countries. Using data from 1071 invasive pathologically confirmed breast cancer cases and 2096 controls from the Ghana Breast Health Study conducted from 2013 to 2015, we investigated associations with mosquito control products to reduce the spread of mosquito-borne diseases, such as malaria. These mosquito control products were insecticide-treated nets, mosquito coils, repellent room sprays, and skin creams for personal protection against mosquitos. Multivariable and polytomous logistic regression models were used to estimate odds ratios (OR_adj_) and 95% confidence intervals (CI) with breast cancer risk-adjusted for potential confounders and known risk factors. Among controls, the reported use of mosquito control products were mosquito coils (65%), followed by insecticide-treated nets (56%), repellent room sprays (53%), and repellent skin creams (15%). Compared to a referent group of participants unexposed to mosquito control products, there was no significant association between breast cancer risk and mosquito coils. There was an association in breast cancer risk with reported use of insecticide-treated nets; however, that association was weak and not statistically significant. Participants who reported using repellent sprays were at elevated risks compared to women who did not use any mosquito control products, even after adjustment for all other mosquito control products (OR = 1.42, 95% CI=1.15–1.75). We had limited power to detect an association with repellent skin creams. Although only a few participants reported using repellent room sprays weekly/daily or < month-monthly, no trends were evident with increased frequency of use of repellent sprays, and there was no statistical evidence of heterogeneity by estrogen receptor (ER) status (*p-*het > 0.25). Our analysis was limited when determining if an association existed with repellent skin creams; therefore, we cannot conclude an association. We found limited evidence of risk associations with widely used mosquito coils and insecticide-treated nets, which are reassuring given their importance for malaria prevention. Our findings regarding specific breast cancer risk associations, specifically those observed between repellent sprays, require further study.

**Supplementary Information:**

The online version contains supplementary material available at 10.1186/s13058-023-01737-x.

## Background

Breast cancer incidence is increasing in many countries in sub-Saharan Africa (SSA), with about half of the cases diagnosed among women less than 50 years of age [1–3]. The Ghana Breast Health Study is one of a handful of molecular epidemiologic studies in SSA aimed at identifying possible risk factors that might be amenable to preventative efforts [1, 4]. Malaria is a significant public health challenge, with roughly 90% of all malaria deaths occurring in SSA [5]. Some mosquito control products contain insecticides and remain an essential tool in eliminating the transmission of the parasites responsible for causing malaria in humans. Of concern, however, is whether the constituents of these insecticides might have adverse effects on breast cancer risk, particularly insecticides with endocrine-disrupting activities [6]. Given long-standing programs to tackle the high mortality burden of malaria in Ghana, investigations regarding the association between mosquito control products with presumed insecticide exposure and breast cancer risk are critically needed [7, 8], with few high-quality population-based epidemiologic studies having been conducted.

The most commonly used measures for mosquito control include indoor residual spraying, insecticide-treated nets, or a combination thereof [9]. There has been extensive debate about the effects of indoor residual spraying on women’s health [10], with a significant concern being that persistent organochlorine insecticides such as dichlorodiphenyltrichloroethane (DDT) can mimic estrogen and bind estrogen receptors, possibly increasing the risk of estrogen-mediated breast cancers [11]. In addition to indoor residual spraying and insecticide-treated nets, other commercially available anti-mosquito interventions that have been increasingly used in Ghana in recent years include mosquito coils, long-lasting repellent sprays, and skin creams [12]. Most mosquito repellents sold over the counter are non-biodegradable synthetic chemicals, which may lead to environmental and unacceptable health risks [13].

As malaria coexists with breast cancer in Ghana, we aimed to determine if the reported use of different types of mosquito control products with possible insecticide exposures was associated with breast cancer risk in the Ghana Breast Health Study [1].

## Methods

### Study population

We used data from the Ghana Breast Health Study (GBHS), a multi-disciplinary population-based case–control study conducted from 2013 to 2015 in two major cities of Ghana: Accra and Kumasi. Participant characteristics and methods of screening and recruitment have been described in detail previously [1, 14, 15]. In brief, the study enrolled eligible cases who were recommended for a breast biopsy for a suspicious malignant lesion or who presented for pathologically confirmed breast cancer treatment in the previous year at one of three primary cancer treatment facilities [Korle Bu Teaching Hospital (KBTH), Komfo Anokye Teaching Hospital (KATH), and Peace and Love Hospital (PLH)]. For this analysis, we focused on 1071 pathologically confirmed invasive breast cancer cases and 2096 frequency-matched population-based controls who were identified using census-based sampling. All study participants (cases and controls) had to meet the following criteria: (1) female; (2) between the ages of 18 and 74; (3) lived in one of the 22 defined municipal districts for at least a year prior to enrollment; and (4) completed an in-person interview in English or Twi language.

Controls were frequency-matched to cases based on the area of residence and were cancer-free at the time of recruitment. Hospital data from 2010 to 2012 were used to identify enumeration areas with the highest concentration of breast cancer patients to frequency match controls to cases according to location [15]. The areas chosen for inclusion included those where potential controls could readily travel to the three study hospitals (KBTH, KATH, and PLH). Controls were frequency-matched to cases on age and residence districts in Accra and Kumasi. Participants voluntarily participated in the GBHS, giving oral informed consent for an in-person interview and the collection of blood, saliva, and stool specimens. Response rates to interviews were 99.2% in cases and 91.9% in controls, with the vast majority (82–99%) of interviewed subjects providing blood and saliva samples [1].

### Questionnaire data for exposure assessment

Previous studies have evaluated the questionnaire data and reported on associations of breast cancer risk with education, reproductive factors, body size, and family history of breast cancer [1, 2, 16]. The questionnaire also asked questions regarding whether malaria vector control products had ever been used and included (yes/no) questions on the use of insecticide-treated mosquito coils, nets, repellent room sprays, repellent skin creams, and other mosquito control products. The questionnaire also asked about the frequency of use for each anti-mosquito intervention: daily, weekly, monthly, or more than once a month.

### Statistical analysis

Data analysis was conducted using Stata MP14.2 (College Station, TX). Chi-squared tests were used to estimate associations with anti-mosquito control products by case–control status and determine associations among controls only. We calculated Spearman correlations between different mosquito control products. Odds ratios (OR) and 95% confidence intervals (CI) were estimated using logistic regression to determine associations between breast cancer risk and malaria-related insecticide exposures (ever used a mosquito coil, an insecticide-treated net, repellent room sprays, repellent skin creams, and other mosquito control products). Minimally adjusted models adjusting for matching factors (age and site) were first performed. Fully adjusted variables included age, site, education, age at menarche, body size, number of pregnancies, age at first birth, the median duration of breastfeeding months, menopause, and age at menopause, as previously described [2]. To determine if there was significant heterogeneity by age at diagnosis, we performed likelihood ratio tests (pLRT) with and without an interaction term for age in the model, as previously described [2]. Polytomous logistic regression analyses were used to estimate the risk for tumor estrogen receptor (ER) status. Heterogeneity between anti-mosquito control products was assessed using polytomous logistic regression analyses restricted to cases (case-only analyses) with ER status as the outcome variable, as previously described [2]. All statistical tests were two-sided, and a *p-*value of 0.05 was considered to denote statistical significance.

## Results

A total of 1071 malignant breast cancer cases and 2096 controls were included in the present analysis (Table 1). Cases were slightly older and more likely menopausal than controls, reflecting that controls were initially frequency-matched to all women suspected of breast cancer, and some women who were generally younger were subsequently diagnosed with benign breast disease. Cases, on average, had more years of formal education than controls; 375 (37%) reached senior secondary school compared to 512 (25%) of controls. Additionally, cases more often than controls reported later ages of menarche, fewer births, later ages at first birth, and lower median breastfeeding months and years.

**Table 1:** Descriptive characteristics of 1071 invasive breast cancer cases and 2096 controls and mosquito control methods in the Ghana Breast Health Study

Study characteristics	Controls		Cases	
	2096	%	1071	%
Age				
< 35	435	21	107	10
35–44	561	27	265	25
45–55	554	26	312	29
55+	546	26	387	36
Recruitment city				
Accra	728	35	374	35
Kumasi	1368	65	697	65
Education				
No formal education	498	24	238	24
Primary school	369	18	146	15
Junior secondary school	654	32	245	24
> Senior secondary school	512	25	375	37
Unknown	73		67	
Family history of breast cancer				
No	2036	98	985	93
Yes	46	2	72	7
Unknown	24		14	1
Age at menarche (years)				
< 15	568	30	258	28
15	548	29	238	26
16	383	20	212	23
> 17	395	21	213	23
Unknown	212		150	
Body size				
Slight	585	29	243	24
Average	827	40	413	41
Slightly heavy	470	23	249	25
Heavy	163	8	99	10
Unknown	61		67	
Parity				
Median among parous (IQR)	4 (2–5)		3 (2–5)	
Nulliparous	228	11	101	9
1–2	528	25	302	27
3–4	683	32	349	31
> 5	649	31	315	28
Unknown	8		4	
Age at first birth				
Median years (IQR)	20 (18–24)		21 (19–25)	
< 18	552	31	229	25
19–21	509	28	253	28
22–25	411	23	244	27
26+	322	18	190	21
Breastfeeding years (among parous women)				
Median years (IQR)	5 (3.0–8.0)		4.4 (2.6–7.5)	
Never	31	2	165	16
< 1	47	3	41	4
1– < 3	360	20	184	17
3– < 5	440	24	246	23
5– < 10	618	34	299	28
≥ 10	321	18	117	11
Unknown	61		60	
Median breastfeeding/pregnancy (months)				
Median months (IQR)	18 (15–24)		18 (12–24)	
≤ 12	352	20	232	26
13–18	692	39	333	38
> 18	747	42	320	36
Unknown	87		144	
Menopausal status				
Premenopausal	1276	61	468	44
Postmenopausal	816	39	601	56
Unknown	14		2	
Age at menopause				
Median years (IQR)	49 (45–51)		49 (45–51)	
< 45	119	18	81	17
45–49	222	33	156	33
50–54	267	39	183	39
≥ 55	68	10	53	11
Unknown	147		130	
Insecticide-treated net				
No	909	44	484	45
Yes	1175	56	581	55
Unknown	12		6	
Repellent room spray				
No	980	47	398	37
Yes	1101	53	667	63
Unknown	15		6	
Repellent skin cream				
No	1750	85	867	82
Yes	321	15	188	18
Unknown	25		16	
Mosquito coil				
No	736	35	419	39
Yes	1344	65	644	61
Unknown	16		8	
Other mosquito control products				
No	1995	98	1028	99
Yes	32	2	13	1
Unknown	69		30	
Single use mosquito control product categories				
No reported use of any insecticide vector control product	191	9	77	7
Ever use insecticide-treated net only	218	10	117	11
Ever use repellent room spray only	131	6	110	10
Ever use repellent skin cream only	10	0	3	0
Ever use mosquito coil only	216	10	78	7
Use of multiple mosquito control products	1330	63	686	64
Any combination use of mosquito control products				
Ever use any combination of insecticide-treated net with other insecticide products	1175	56	581	54
Ever use any combination of repellent room spray with other insecticide products	1101	53	667	63
Ever use any combination of repellent skin cream with other insecticide products	321	15	188	18
Ever use any combination of mosquito coil with other insecticide products	1344	65	644	61

*N* of subjects may not total due to missing values

Among controls, the most commonly used malaria vector control products were mosquito coils (65%), followed by insecticide-treated nets (56%), repellent room spray (53%), and repellent skin cream (15%). Overall, 191 (9%) controls and 77 (7%) cases were not exposed to any anti-malaria control product. Only 32 participant controls (2%) reported using other mosquito control products, suggesting that we captured the vast majority of mosquito control intervention types.

The reported single use of two mosquito control products between cases and controls was similar. Among the combination of different mosquito control products, ever use of any combination of repellent room spray with other insecticide products was higher among cases (63%) than controls (53%), and ever use of any combination of mosquito coil was higher among controls (65%) than cases (61%).

Frequency of all possible combinations of use of mosquito control products among controls is shown in Additional file 1: Table S1. Among controls, the combination of insecticide-treated nets, repellent room spray, and mosquito coils was the most common (17%), followed by insecticide-treated nets and mosquito coils (12%) and insecticide spray and mosquito coils (11%). There were significant correlations between ever use of the different anti-mosquito control products, but Rho was < 0.25 for all of the different combinations (Additional file 2: Table S2). Examination of the anti-mosquito products among the controls by study risk factors found differences by age, study city, education, body size, and reproductive factors (parity and age at first birth) but not family history of breast cancer or age of menarche (Additional file 3: Table S3).

Table 2 shows the results of multivariable models investigating the potential associations between breast cancer and exposure from different malaria vector control products. In both the partially adjusted (for age and site) and the multivariable models, when we compared women who reported the use of either one of each mosquito control product compared to those who did not use the individual product, there was no association with breast cancer risk for reported use of insecticide-treated nets [OR_adj_ = 1.10 (95% CI=0.93–1.29), *p* = 0.25], mosquito coils [OR_adj_ = 0.98 (95% CI=0.82–1.16), *p* = 0.77], or other mosquito control products [OR_adj_ = 0.87 (95% CI=0.44–1.70), *p* = 0.68]. In the partially adjusted model, we observed increases in breast cancer risk with the use of both repellent room sprays and skin creams [respective ORs = 1.80 (95% CI=1.53–2.11) and OR = 1.51 (95% CI=1.23–1.87), *p* < 0.001]. In the multivariable model (adjusting for age, site, education, body size, age at menarche, number of pregnancies, age at first birth, median breastfeeding, menopausal status, and age at menopause), the ORs for repellent room sprays and skin creams were attenuated but remained statistically significant for repellent room sprays [OR_adj_ = 1.56 (95% CI=1.32–1.84)] and for repellent skin creams [OR_adj_ = 1.35 (95% CI=1.08–1.67)].

**Table 2:** Association results of mosquito control methods and breast cancer risk in the Ghana Breast Health Study

Study characteristics	Controls	Cases	OR	95%	CI	*p*	OR_adj_	95%	CI	*p*
Insecticide-treated net										
Never use	909	484	Ref				Ref			
Ever use	1175	581	1.06	0.91	1.24	0.43	1.10	0.93	1.29	0.25
Repellent room spray										
Never use	980	398	Ref				Ref			
Ever use	1101	667	1.80	1.53	2.11	6.01e−13	1.56	1.32	1.84	2.59e−07
Repellent skin cream										
Never use	1750	867	Ref				Ref			
Ever use	321	188	1.51	1.23	1.87	0.00009	1.35	1.08	1.67	0.007
Mosquito coil										
Never use	736	419	Ref				Ref			
Ever use	1344	644	0.99	0.84	1.16	0.88	0.98	0.82	1.16	0.77
Other Mosquito control products										
Never use	1995	1028	Ref				Ref			
Ever use	32	13	0.87	0.45	1.68	0.68	0.87	0.44	1.70	0.68

OR adjusted for age and site

OR_adj_ models adjusted for age, site, education, body size, age at menarche, number of pregnancies, age at first birth, median breastfeeding, menopausal status, and age at menopause

We also assessed reported daily, weekly, monthly, and more than monthly use (Additional file 4: Table S4), which did not reveal any trends with different anti-mosquito control products, although numbers for this analysis became sparse.

Many participants used multiple products in combination (Additional file 3: Table S3). To obtain a better picture of associations, given the possible overlap in the active ingredients included in the various mosquito control products, we examined the associations using a clean reference group of women with no reported use of any mosquito vector control product (Table 3). The use of only mosquito coils, similar to the previous analysis, did not show an association with risk (Table 3). We observed the use of only repellent room spray significantly associated with risk [OR_adj_ = 1.85 (95% CI=1.25–2.72), *p* = 0.002], although the use of insecticide-treated nets alone was also elevated [OR_adj_ = 1.39 (95% CI 0.96–2.01)]; it was not statistically significant. Due to the limited number of cases, we could not evaluate the single use of repellent skin creams alone and their association with breast cancer risk. Mutually adjusting for the use of the different mosquito control products attenuated the associations for all products, with only repellent spray remaining statistically significant compared to those not reporting any mosquito control products [OR_madj_ = 1.42 (95% CI=1.15–1.75), Table 3].

**Table 3:** Association results of mosquito control methods alone or in combination with other products compared to clean referent with and without adjustment for other control products

Exposure	Controls	Cases	OR_adj_	95%	CI	*p*	OR_madj_	95%	CI	*p*
Never reported use of any insecticide vector control product	191	77	Ref							
Ever use insecticide-treated net only	218	117	1.39	0.96	2.01	0.079				
Ever use repellent room spray only	131	110	1.85	1.25	2.72	0.002				
Ever use repellent skin cream only	10	3	–							
Ever use mosquito coil only	216	78	0.97	0.66	1.43	0.873				
Never reported use of any insecticide vector control product	191	77	Ref				Ref			
Ever use any combination of insecticide-treated net with other insecticide products	1175	581	1.47	1.09	1.99	0.01	1.19	0.86	1.65	0.29
Ever use any combination of repellent spray with other insecticide products	1101	667	1.74	1.29	2.36	< 0.001	1.42	1.15	1.75	0.001
Ever use any combination of repellent cream with other insecticide products	321	188	1.83	1.29	2.59	0.001	1.21	0.97	1.52	0.08
Ever use any combination of mosquito coil with other insecticide products	1344	644	1.39	1.02	1.87	0.04	0.83	0.58	1.18	0.30

OR_adj_ adjusted for age, site, education, body size, age at menarche, number of pregnancies, age at first birth, median breastfeeding, menopausal status, and age at menopause

OR_madj_ adjusted for age, site, education, body size, age at menarche, number of pregnancies, age at first birth, median breastfeeding, menopausal status, and age at menopause and all mosquito control products

To determine if there might be a potential cohort effect, we stratified by age at diagnosis/recruitment and examined associations (Additional file 5: Table S5). Although numbers became sparse, we did not observe significant associations. We observed a positive association for age groups < 60 between breast cancer risk and the use of only insecticide-treated nets compared to no reported use of any insecticide vector control product. The strongest association with risk was seen for those reporting using only repellent room sprays among women aged 40–49 [OR = 2.38, (95% CI= 1.19–4.77), *p* = 0.01]. There was no association between the use of only mosquito coils and breast cancer risk in any age group when compared to no reported use of any insecticide vector control product and a marginal suggestion of differences by age (pLRT = 0.05). There was also a marginal suggestion of differences by age for mosquito coil with an inverse association among the older age groups (50+, Additional file 5: Table S5).

To determine if there might be differences in associations by ER status, potentially pointing to a hormonal effect, we estimated breast cancer risk by ER status of tumors (ER-positive, *n* = 390 and ER-negative, *n* = 380, Table 4). We did not observe any significant heterogeneity by ER status (Table 4). Although based on few participants, the association with repellent room sprays compared to the never-reported use of any mosquito control products was higher for ER-positive tumors (*N* = 44) compared to ER-negative (*N* = 35) tumors, although the test for heterogeneity was not significant (ER-positive OR = 2.41, 95% CI=1.36–4.29, *p* = 0.003; ER-negative OR = 1.59, 95% CI=0.91–2.78, *p* = 0.10; *p*-het = 0.25). The association of any reported repellent room sprays and mosquito coils compared to no mosquito control products showed no evidence of heterogeneity (*p*-het > 0.19).

**Table 4:** Association results of mosquito control methods alone or in combination with other products compared to clean referent with and without adjustment for other control products by estrogen receptor (ER) tumor receptor expression status

	Controls	ER-positive	ER-negative	ER-positive *N* = 390			*p*	ER-negative *N* = 380			*p*	*p*-het
				OR_1_	95%	CI		OR_1_	95%	CI		
Use of clean referent group and insecticide exposures												
Never reported use of any insecticide vector control product	191	23	30	Ref				Ref				
Ever use insecticide-treated net only	218	35	51	1.35	0.75	2.41	0.31	1.41	0.85	2.35	0.19	0.90
Ever use repellent room spray only	131	44	35	2.41	1.36	4.29	0.003	1.59	0.91	2.78	0.10	0.25
Ever use repellent skin cream only	10	1	2	–								
Ever use mosquito coil only	216	25	20	1.08	0.58	1.99	0.81	0.58	0.31	1.07	0.08	0.13

				OR_2_	95%	CI	*p*	OR_2_	95%	CI	*p*	*p*-het
Never reported use of any insecticide vector control product	191	23	30	Ref				Ref				
Ever use any combination of insecticide-treated nets with other insecticide products	1175	214	216	1.40	0.83	2.36	0.20	1.04	0.64	1.67	0.88	0.36
Ever use any combination of repellent spray with other insecticide products	1101	257	242	1.79	1.37	2.33	2.00E−05	1.80	1.38	2.35	2.00E−05	0.97
Ever use any combination of repellent cream with other insecticide products	321	72	71	1.34	0.99	1.84	0.062	1.35	0.99	1.84	0.06	0.98
Ever use any combination of mosquito coil with other insecticide products	1344	238	223	0.95	0.74	1.24	0.724	0.77	0.59	1.00	0.05	0.19

OR_1_ Polytomous logistic regression models were adjusted for age, education, study site, body size, family history of breast cancer, menopausal status, and age at menopause. Abbreviations: *CI,* confidence interval; *ER,* estrogen receptor; *OR,* odds ratio; *p-het, p*-heterogeneity test

OR_2_ Polytomous logistic regression models were adjusted for age, education, study site, body size, family history of breast cancer, menopausal status, age at menopause, and all other mosquito control products. Abbreviations: *CI,* confidence interval; *ER,* estrogen receptor; *OR,* odds ratio; *p-het, p*-heterogeneity test

## Discussion

Malaria and breast cancer are co-occurring public health burdens in Ghana, and it is currently unknown whether commonly used anti-mosquito interventions are associated with breast cancer risk. In this population-based case–control study, we examined whether different mosquito control exposures were associated with breast cancer risk among Ghanaian women. Among our study participants, mosquito coils, insecticide-treated nets, and repellent sprays were prevalent. We observed no association between mosquito coils and breast cancer risk. Although we observed a positive association between insecticide-treated nets and breast cancer risk, it was not statistically significant. However, the associations with repellent room sprays require further investigation.

Similar to other reports [17], we found that mosquito nets and coils were the most common mosquito control methods used in Ghana, supporting that our data focused on a representative sample of the Ghanaian population. From the 2019 Ghana Malaria Indicator report, 52% of households reported at least one insecticide-treated net for every two persons, and 67% of insecticide-treated nets were obtained from mass distribution campaigns in 2018 [18].

There was no evidence of any substantial association of breast cancer risk with the use of insecticide-treated nets in our study. Pyrethroids are the primary insecticides used for insecticide-treated nets because they are cost-effective, long-lasting, and a readily accessible preventive intervention against malaria [19, 20]. Additionally, the WHO recommends insecticide-treated nets for malaria vector control, as they serve as personal protection and remain a viable, safe option. Insecticide exposure from insecticide-treated nets is likely dermal exposure, which may be a lessened risk compared to inhalation. Our findings’ lack of association between insecticide-treated nets and breast cancer is encouraging, with global public health implications favoring continued deployment in malaria-endemic areas.

In our study, the reported use of mosquito coils was not associated with breast cancer risk. Mosquito coils have gained popularity in general use in Ghana as they effectively repel mosquitoes, are inexpensive, and are accessible to lower-income families, even though they are not a WHO-approved method [21, 22]. Natural plant-based repellents such as the oil of citronella are one method used in mosquito coils to repel mosquitos, but increasingly synthetic chemical repellents are being used in these products [23]. Synthetic chemical repellents may present a risk to human health due to frequent indoor use trapping chemical particles inside homes [24]. While we found limited to no evidence of an association with breast cancer risk, there are concerns that these products may have other health risks, as studies have shown that the burning of coils emits heavy metals, aldehydes, and polycyclic aromatic hydrocarbons [12, 17, 25].

Commercially available insecticide-repellent room sprays and long-lasting repellent skin creams are other commercially available products that supersede conventional techniques [12]. In our study, the reported use of repellent room sprays and skin creams compared to participants reporting no use was associated with approximately 50% and 30% increased risk, respectively. However, because creams were used in conjunction with many other products, we had limited ability to determine if they were independently associated with risk. For repellent room sprays, however, we observed elevated risk compared to participants reporting no use of mosquito control products and independent of other products. Repellent room sprays are sprayed indoors, in living or sleeping areas, while skin creams are applied directly to the skin. There were few participants with ever use of repellent skin creams, and we did not observe distinctive differences for the use of either modality (repellent room spray or skin cream) by frequency of use, with similar risks observed for those who used them monthly, weekly, or daily—albeit all based on small numbers. Insecticide-repellent room sprays and topical skin cream repellents are readily available in Ghana and may contain various active compounds to prevent mosquito bites, including N, N-diethyl-m-toluamide (DEET) [12], one of the most effective insect-repellent sprays widely used globally [26]. DEET is a highly effective repellent room spray in preventing mosquito bites. Despite its widespread usage, there are no epidemiological studies that have specifically evaluated potential associations between DEET and breast cancer. A case–control study by Hardell et al. [27] included 148 cases and 314 controls which examined occupational exposures to DEET and their association with testicular cancer, where an elevated risk of testicular cancer was found to be associated with high exposure to insect repellents [27]. In a population-based study, Pahwa et al. [28] found that DEET exposure contributed to non-Hodgkin lymphoma [28]. Although these studies have suggested a potential link between DEET and different types of cancer, there is currently no evidence that DEET is an endocrine disruptor, and the effects on humans following exposure are unknown [29].

In our analyses, we also investigated whether associations differed by ER status, potentially pointing to endocrine mechanisms, but found limited evidence of heterogeneity and had limited numbers to investigate this. A link between malaria insecticides and breast cancer has been shown in early studies examining DDT exposure. DDT is a well-known organochlorine that was once a widely used insecticide used for indoor residual spraying and sprayed extensively in agriculture and populated areas until banned in many countries after being discovered to be an endocrine disrupter [30, 31]. Decades later, several studies using Child Health and Development data have shown that DDT exposure to high levels during childhood through early adolescence may contribute to the risk of breast cancer [32–34]. For instance, in a prospective nested case–control study of young women during 1959–1967, Cohn et al. [34] examined whether DDT exposure in young women during the period of peak DDT use predicts breast cancer [34]. This study found that women who were less than 14 years of age when exposed to DDT experienced a significant fivefold increased risk of breast cancer [34]. Several years later, Cohn et al. [32] conducted a second prospective nested case–control study to investigate whether age at diagnosis modifies the interaction of DDT with age at exposure. Cohn et al. [32] found that women who were first exposed to DDT in infancy more often developed breast cancer before 50 years of age, whereas women who were first exposed to DDT after infancy had a later onset of breast cancer [32]. However, studies that have examined adult exposure to DDT and breast cancer have been inconclusive [35–37].

Epidemiological studies that have examined the potential relationship between insecticide exposure and cancer risk have been inconsistent, particularly as related to breast cancer risk [38]. This is likely because of the complexities of measuring insecticide exposure, timing, and frequency. Besides exposure assessment, studies also need to consider that the term “insecticide,” which encompasses different active ingredients, adds to this complexity. Hence, study inconsistencies in associations between insecticide exposure and breast cancer may be due to differences in the insecticide classes used (organochlorines, organophosphates, carbamates, and pyrethroids) and insecticide practices in different regions. Although we observed an association between repellent room sprays and breast cancer risk, it is unclear whether exposure to other interventions used in malaria control is the specific source of this association. Limited information exists on exposure levels, a critical factor when assessing health effects; therefore, we cannot draw definitive conclusions about the long-term health risks of using anti-mosquito products among Ghanaian women. Potential health issues related to insecticide use are of concern; however, insecticide use may have more positive impacts than negative ones, such as preventing severe malaria infection or death. Even though our study found some limited evidence to link breast cancer risk with exposure to repellent room sprays and skin creams, results were often times based on small numbers and inconsistent findings. Therefore, these exposures are worth monitoring for potential associations in future studies.

Limitations of our current study included a lack of information on the timing and duration of use or exposure to specific active ingredients. Another limitation was a lack of information on whether participants were exposed to insecticides from Ghana’s national program’s two main malaria intervention methods (indoor residual spraying, insecticide-treated nets) or commercially available products on the market (mosquito coils, repellent room sprays, and creams). To clarify whether these easily accessible over-the-counter commercial products may represent a risk for breast cancer, additional research is required to investigate insecticide exposures from these products. Another drawback is the lack of precise assessments of each participant’s insecticide exposure and scant data on other possible exposures and confounding factors that could present alternative explanations for our findings. Of particular concern was the possibility of residual confounding by education since mosquito control products were more frequently reported among those with higher education. Recall bias is another potential limitation of all case–control studies; however, we suspect this to be minimal since we only observed associations for specific types of products. In this study, only 32 controls (2%) reported using other mosquito control products, suggesting that we captured the common ways Ghanaian women are exposed to anti-mosquito-related insecticides.

In conclusion, the lack of association between insecticide-treated nets and mosquito coils with breast cancer in our study findings is encouraging, given their wide use globally for malaria control programs. Further research on specific products and active chemical formulations is needed, especially for repellent sprays, to determine if the risks observed in our study are plausible.

### Supplementary Information

**Additional file 1. Table S1**. Frequency of combinations of mosquito control product use among 2096 controls.

**Additional file 2. Table S2**. Rho (in bold) and p-values from Spearman correlation for mosquito control product use among 2096 controls.

**Additional file 3. Table S3**. Mosquito vector control products among controls by identified study breast cancer risk factors.

**Additional file 4. Table S4**. Association results of mosquito control methods and frequency of use and breast cancer risk in the Ghana Breast Health Study.

**Additional file 5. Table S5**. Association of insecticide exposures and breast cancer risk stratified by age at diagnosis/recruitment.

## Data Availability

The Ghana Breast Health Study dataset is available from the corresponding authors upon request.
