# Supplementary material for: Mosquito control exposures and breast cancer risk: analysis of 1071 cases and 2096 controls from the Ghana Breast Health Study
Source: Breast Cancer Res. 2023 Dec 11;25:150. doi: 10.1186/s13058-023-01737-x (PMC10714652; doi:10.1186/s13058-023-01737-x)

**Additional file 1. Table S1:** Frequency of combinations of mosquito control product use among 2096 controls


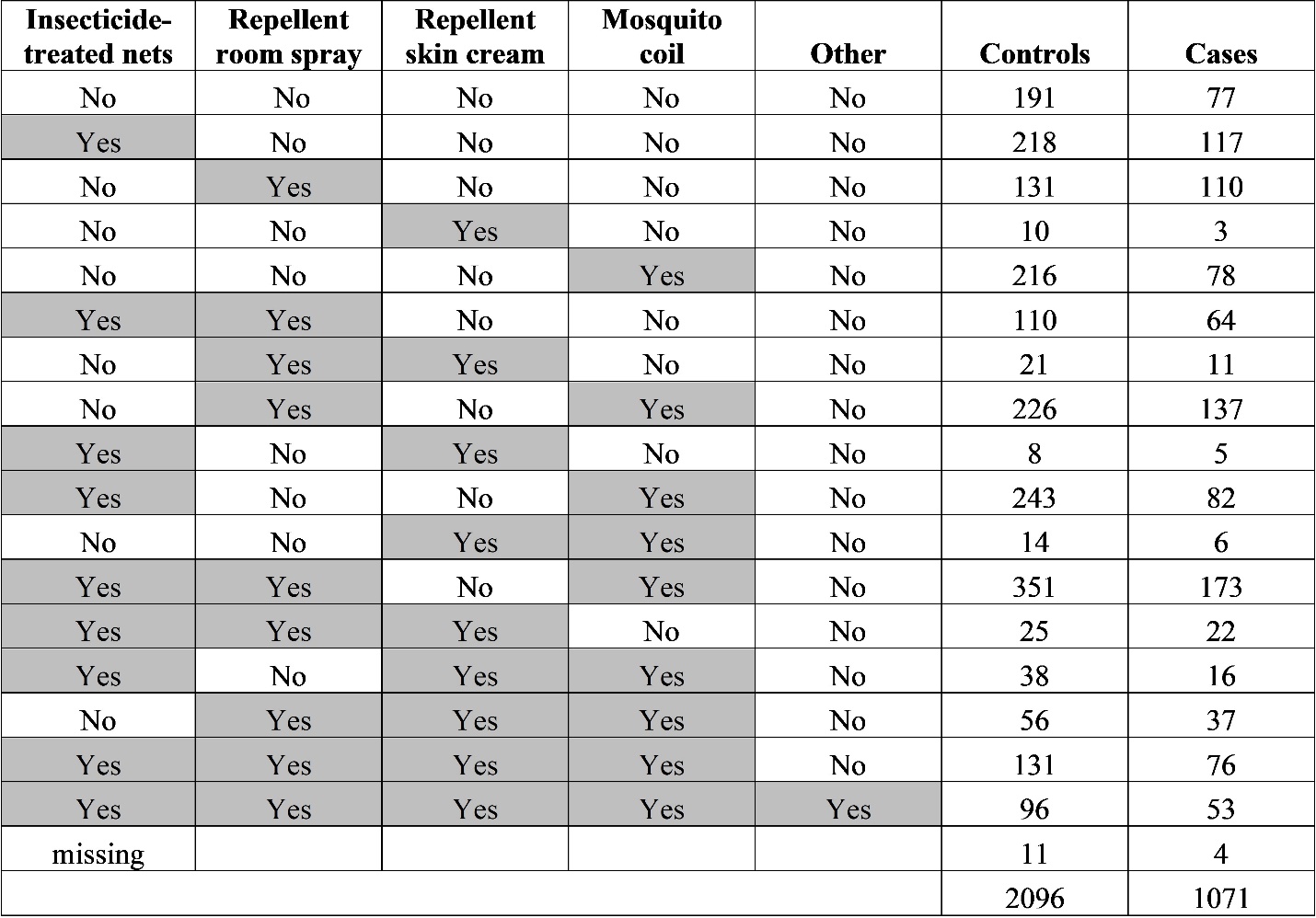

Supplement: Supplementary file 1 — Additional file 1. Table S1. Frequency of combinations of mosquito control product use among 2096 controls. [file 13058_2023_1737_MOESM1_ESM.docx]
