# Supplementary material for: Mosquito control exposures and breast cancer risk: analysis of 1071 cases and 2096 controls from the Ghana Breast Health Study
Source: Breast Cancer Res. 2023 Dec 11;25:150. doi: 10.1186/s13058-023-01737-x (PMC10714652; doi:10.1186/s13058-023-01737-x)

**Additional file 2. Table S2:** Rho (in bold) and p-values from Spearman correlation for mosquito control product use among 2096 controls


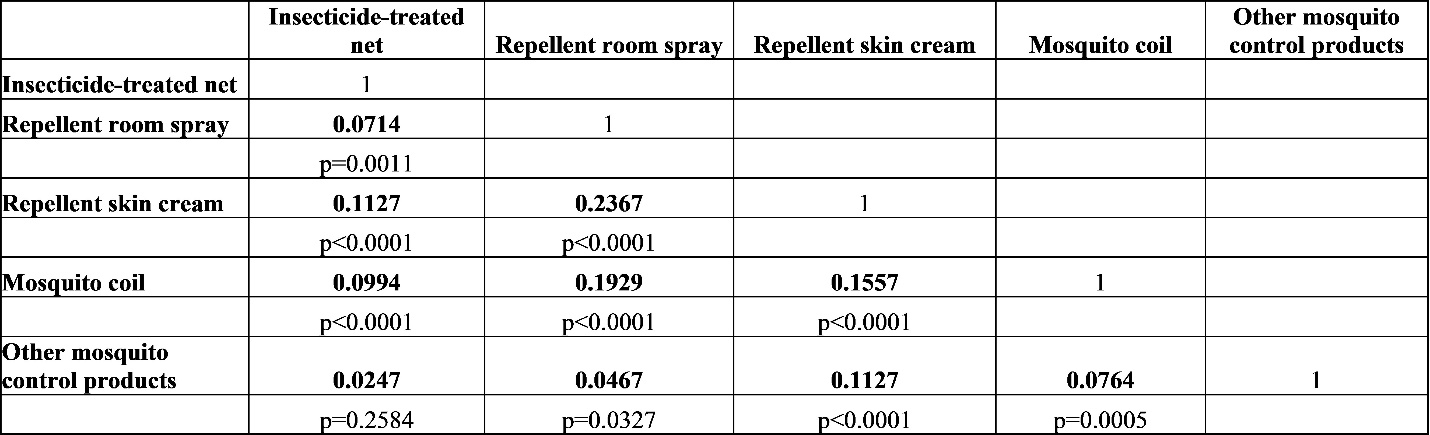

Supplement: Supplementary file 2 — Additional file 2. Table S2. Rho (in bold) and p-values from Spearman correlation for mosquito control product use among 2096 controls. [file 13058_2023_1737_MOESM2_ESM.docx]
