# Supplementary material for: Mosquito control exposures and breast cancer risk: analysis of 1071 cases and 2096 controls from the Ghana Breast Health Study
Source: Breast Cancer Res. 2023 Dec 11;25:150. doi: 10.1186/s13058-023-01737-x (PMC10714652; doi:10.1186/s13058-023-01737-x)

**Additional file 3. Table S3**: Mosquito vector control products among controls by identified study breast cancer risk factors


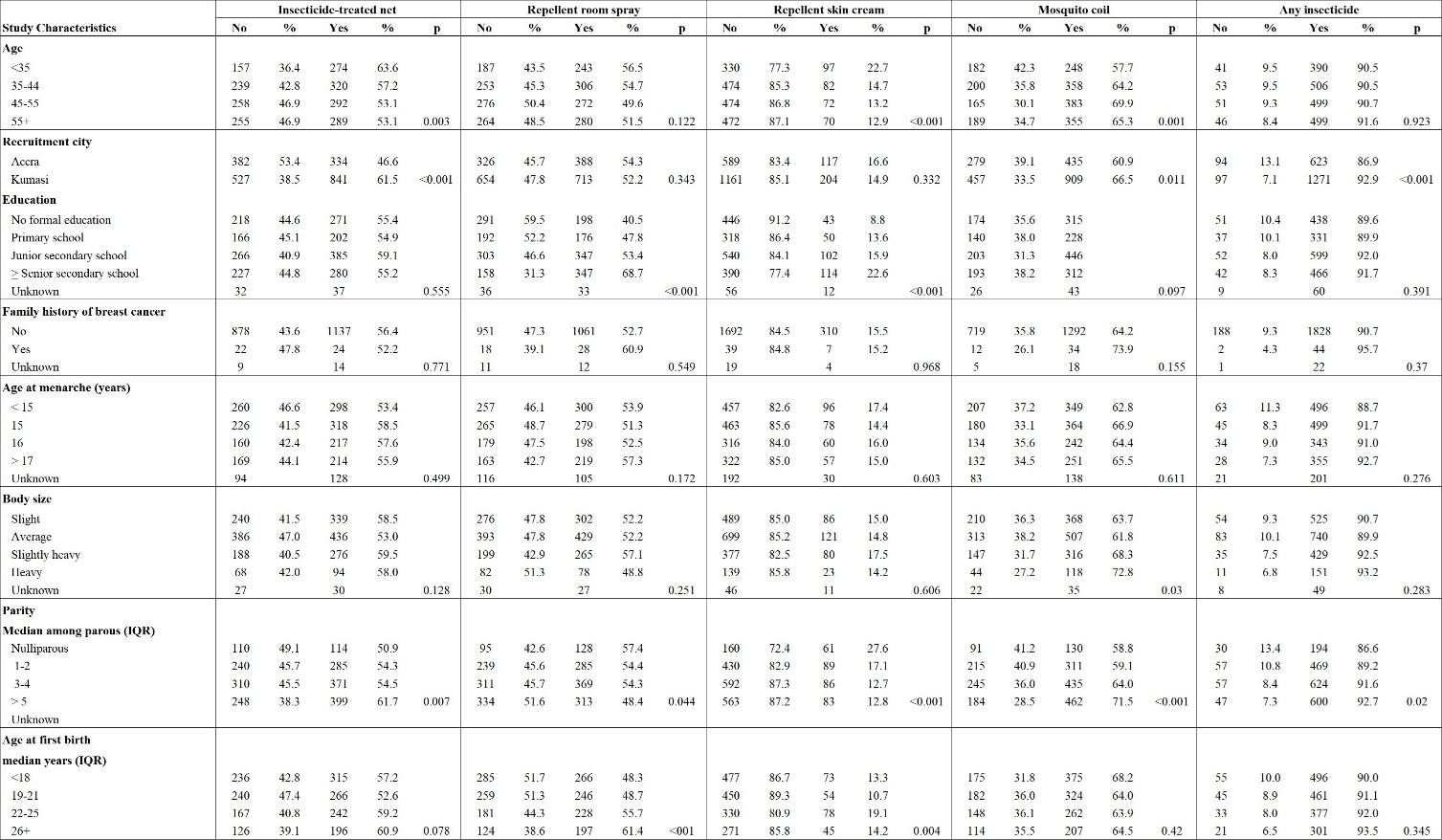

Supplement: Supplementary file 3 — Additional file 3. Table S3. Mosquito vector control products among controls by identified study breast cancer risk factors. [file 13058_2023_1737_MOESM3_ESM.docx]
