# Supplementary material for: Mosquito control exposures and breast cancer risk: analysis of 1071 cases and 2096 controls from the Ghana Breast Health Study
Source: Breast Cancer Res. 2023 Dec 11;25:150. doi: 10.1186/s13058-023-01737-x (PMC10714652; doi:10.1186/s13058-023-01737-x)
Supplement: Supplementary file 5 — Additional file 5. Table S5. Association of insecticide exposures and breast cancer risk stratified by age at diagnosis/recruitment. [file 13058_2023_1737_MOESM5_ESM.docx]

**Additional file 5. Table S5:** Association of insecticide exposures and breast cancer risk stratified by age at diagnosis/recruitment


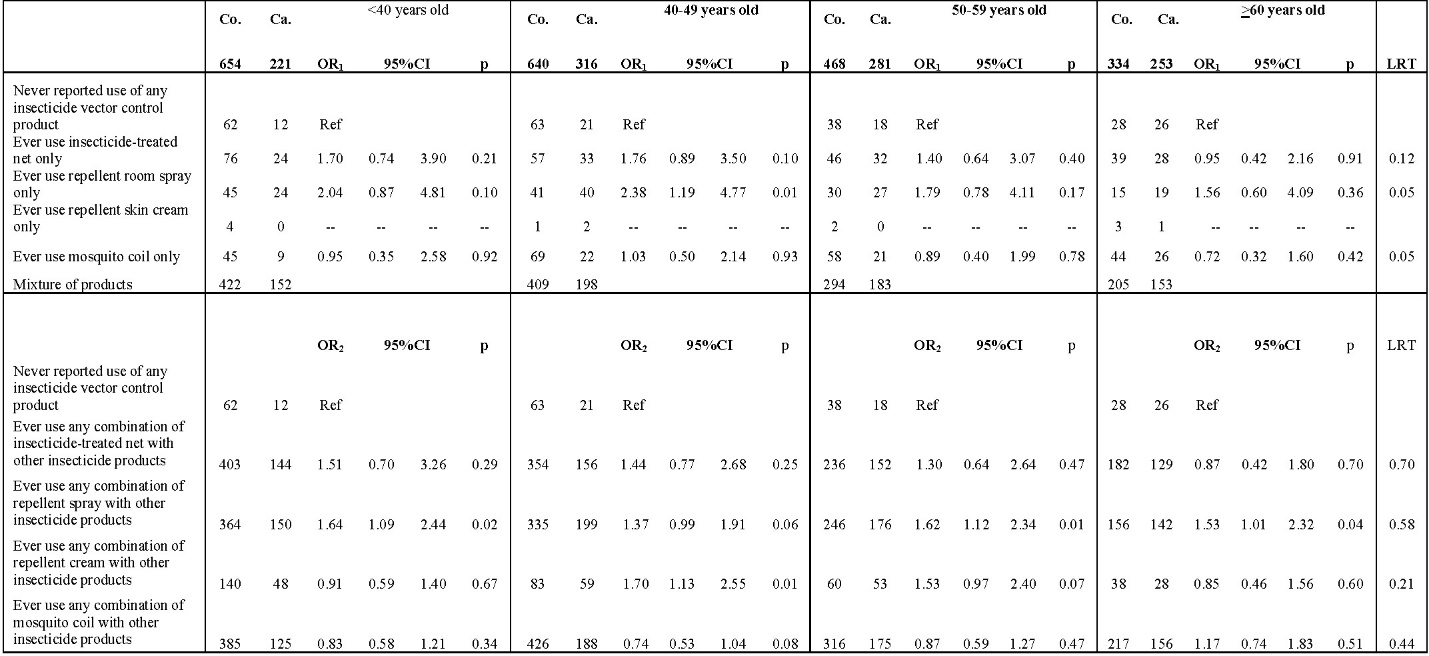


OR_1_=Logistic regression models were adjusted for age, education, study site, body size, family history of breast cancer, menopausal status, and age at menopause. Abbreviations: CI, confidence interval; ER, estrogen receptor; OR, odds ratio; *p*-het, *p*-heterogeneity test. OR_2_=Logistic regression models were adjusted for age, education, study site, body size, family history of breast cancer, menopausal status, age at menopause, and all other mosquito control products.
